# Effects of Sulpiride on True and False Memories of Thematically Related Pictures and Associated Words in Healthy Volunteers

**DOI:** 10.3389/fpsyt.2016.00028

**Published:** 2016-03-18

**Authors:** Regina V. Guarnieri, Rafaela L. Ribeiro, Altay A. Lino de Souza, José Carlos F. Galduróz, Luciene Covolan, Orlando F. A. Bueno

**Affiliations:** ^1^Department of Psychobiology, Universidade Federal de São Paulo, São Paulo, Brazil; ^2^Associação Fundo de Incentivo à Pesquisa, São Paulo, Brazil; ^3^Department of Physiology, Universidade Federal de São Paulo, São Paulo, Brazil

**Keywords:** dopamine, dopamine D_2_ receptor, sulpiride, true recognition, false recognition, amygdala

## Abstract

Episodic memory, working memory, emotional memory, and attention are subject to dopaminergic modulation. However, the potential role of dopamine on the generation of false memories is unknown. This study defined the role of the dopamine D_2_ receptor on true and false recognition memories. Twenty-four young, healthy volunteers ingested a single dose of placebo or 400 mg oral sulpiride, a dopamine D_2_-receptor antagonist, just before starting the recognition memory task in a randomized, double-blind, and placebo-controlled trial. The sulpiride group presented more false recognitions during visual and verbal processing than the placebo group, although both groups had the same indices of true memory. These findings demonstrate that dopamine D_2_ receptors blockade in healthy volunteers can specifically increase the rate of false recognitions. The findings fit well the two-process view of causes of false memories, the activation/monitoring failures model.

## Introduction

Memory has a reconstructive nature, and new details of an experience can be incorporated into a memory trace during reconsolidation. During this process, the details can be forgotten, become distorted, or inaccurate (1). Moreover, in some cases, the memory can be totally false (2). Recognition tests involve two separate process, one often referred as remember response and characterized by recollective experience (episodic memory) and the other one due to a phenomenological experience of familiarity, in which the item is not consciously remembered to have been seen (1, 3).

Over the past decade, research conducted in the mental health field and in the legal field has suggested that emotion may play a role in the production of false memories. In these areas, the phenomenon of false memories has drawn attention because some studies have indicated that certain psychotherapeutic techniques, which are based on the recovery of emotional memories from childhood, can produce vivid memories of events that actually did not occur, such as alleged cases of sexual violence suffered in childhood (4). In the legal area, the impact of emotion on memory function may compromise the exercise of justice, as the person who witnessed a crime, offense, and/or has been the victim of violence may be subject to distortions in their memories (5).

False recognition, “the mistaken belief that one has previously encountered a novel item” as defined by Clancy et al. (6), is usually studied with versions of the Deese–Roediger–McDermott (DRM) paradigm, in which subjects study lists of words that are also related to a non-studied word, a lure that subjects often falsely recall or recognize as having been presented in the study list (7, 8). These authors state that at least two factors determine the likelihood by which a given studied material yields a true or false recollection, namely (a) an associative activation that spreads through the semantic system to non-studied items and (b) a monitoring failure by which distinctive features of non-studied items are not recognized (8).

To evaluate false recognition for visual stimuli, pictures from the International Affective Picture System (IAPS) (9, 10), a widely used database that contains hundreds of complex realistic pictures, were organized in a way to facilitate false memory production in a task called DRM–IAPS (11). The emotional reaction to any stimulus (e.g., images and words) can be classified according to valence and arousal through the test named Self-Assessment Manikin (SAM) (12). The stimuli that trigger emotional reactions with low valence are described as negative, those with medium values of valence are described as neutral, and those with high valence are described as positive. It is also valid for the classification of arousal, when low levels of stimuli are classified as relaxed, average levels are classified as neutral, and high levels are classified as excited (10, 13).

It has long been observed that emotion enhances episodic memory performance (14–17). The effect of the emotional content of stimuli on true memories was shown in a study, in which emotional words were more vividly remembered than neutral ones (18). Emotion promotes better true memory performance compared to neutral events, probably due to increased attention, longer reverberation, better encoding, and elaboration of emotionally loaded stimuli (16, 19). High arousal words and photographs with positive or negative valence have a higher probability of being correctly retrieved compared to similar stimuli classified as neutral (18, 20, 21).

On the other hand, the investigation of the influence of emotion on false memories has yielded some contradictory results. Pesta et al. (22) as well as Kensinger and Corkin (19) have found a decrease in false recognition of emotional events compared to neutral ones. Emotional salience would provide greater distinctiveness to the lures making them less likely to be confounded with true event, thus reducing the incidence of false memories. Otherwise, it has been reported that negative events increase the incidence of false memories to the highest level, neutral events to an intermediate level, and positive stimuli to the lowest level, probably due to a monitoring failure to suppress erroneous acceptance (23). It was also reported (24) that emotionally valenced words increase the tendency of false recollection of unstudied items (lures) due to their level of semantic cohesion. Similarly, it was suggested (25) that emotional load increases semantic relatedness, which in turn may contribute to increased false recollection. It has been reported (26) that emotional words (lures) elicited more positive event-related potentials than did neutral words, and the emotional distractors were falsely recognized more often than the neutral ones. Taken together, these results point to the idea that emotional stimuli are more salient and/or more semantically related than neutral stimuli, and these characteristics form the basis for false recognition effects.

Memory-impairing drugs, such as benzodiazepines [diazepam (27), triazolam (28), lorazepam (27), and alcohol (29–31)], and the anticholinergic drug scopolamine (32) have variable effects on false memory assessed by similar versions of the DRM task (33). Some of these drugs reduced false recognition of related and unrelated lures, while others did not affect false recognition of unrelated lures, and others had the opposite effect. In fact, the memory-enhancer dextroamphetamine (AMP) and the memory-impairing Δ-9-tetrahydrocannabinol (Δ-9-THC) affected false memory in comparison to placebo, although they had opposite effect on true memory, and AMP increased false recognition compared to Δ-9-THC (33).

In spite of a wide literature about dopaminergic modulation of working memory and executive functions (34–36), few studies were aimed to evaluate the role of the dopaminergic system on episodic memory in healthy subjects (37–41). Activation of the dopaminergic system enhances learning and memory formation (38, 41). There is some evidence that blocking dopamine D_2_ receptors impairs declarative memory (40). Plenty of work, both in animals and humans, recognize that the dopaminergic system plays a regulatory role for emotional, motivational, cognitive, and executive functions (42–44). More recently, the role of the dopaminergic system in the process by which the frontal lobe and the striatum control decision-making processes was described (45). Identification of a prior occurrence that happened during recognition depends on decision-making processes.

There is evidence that dopamine modulates the responses of the amygdala to sensory information in Parkinson’s disease patients (46), a finding consistent with findings in experimental animals (47).

We are not aware of studies that aimed to investigate the dopamine D_2_ receptors blockade effects on false memory. If D_2_ dopaminergic receptors modulate the amygdala response to emotional stimuli or either is involved in frontal executive and working memory, we predict that blocking the dopaminergic D_2_ receptor system would affect false recognition. As yet, there is no evidence of the role of a D_2_ antagonist on false memories; here, we evaluate the effects of sulpiride, a selective dopamine D_2_ receptor antagonist on two emotional visual (DRM–IAPS) and verbal (DRM) tasks.

## Materials and Methods

### Participants

The subjects were healthy young male university students. Females were excluded from the study due to the possible side effects of sulpiride, such as galactorrhea with hyperprolactinemia, gynecomastia, breast tenderness, and menstrual irregularity, caused by the action of the drug in the tuberoinfundibular pathway. They were also excluded due to greater emotional variability than men caused by hormonal fluctuations. The subjects were non-smokers with no history of drug abuse, alcoholism, and neurological, psychiatric, or sleep disorders. The subjects’ body mass indices (BMIs) were within the normal range (18–25 kg/m^2^). They were also not on prescription drugs. The participants were matched according to age, education (years of schooling), and intelligence quotient (IQ) measured with the Raven’s Progressive Matrices (48).

The study was approved by the local Ethics Committee (No. CEP 2020/09), and all participants provided written consent according to the Ethics Committee of the Universidade Federal de São Paulo.

### Design and Materials

Twenty-four healthy, young male volunteers were orally administered sulpiride, 400 mg (*n* = 12) or placebo (lactose; *n* = 12).

### Procedure

One week before the experiment, the participants completed the Raven’s Matrices tasks from the State-Trait Anxiety Inventory (STAI) (49, 50) as well as physical, clinical, and psychiatric questionnaires. At the screening, seven candidates were excluded and replaced by other subjects (two had high anxiety levels, two had high BMI, two had low IQ scores, and one subject was a smoker). On the day of the experiment, the volunteers ate a controlled breakfast (free of tryptophan, tyrosine, and caffeine) and then completed the STAI. Following the test instructions, the volunteers ingested the capsules at 7:45 a.m. The memory tasks were performed 3 h after capsule ingestion (sulpiride serum peak). This is the time window necessary to afford sensitivity to cognitive modulation (51). The participants returned home after completing the memory task by taxi.

### Mood Task

The STAI verified the subjects’ baseline anxiety levels (scores above 40 were excluded) 1 week before the experiment and again on the day of the experiment at the beginning (T_1_) and the end (T_2_) of the trial.

### Affective Rating Scale Self-Assessment Manikin

The SAM is a non-verbal pictorial assessment technique that directly measures the pleasure, arousal, and dominance associated with a person’s affective reaction to a wide variety of stimuli. It is an instrument used for subjective evaluation of emotions (12). The person subjectively evaluates the stimuli as to the valence (pleasure–displeasure) and arousal (relaxing–exciting) dimensions immediately after encoding each photo from 20 blocks (6 photos/block) during the study phase (encoding) of the DRM–IAPS task (see below). The participant should select the SAM man doll representing their subjective perception (greater or lesser extent) referring to visual stimuli presented.

The representation of the dimension of *pleasure–displeasure* of SAM ranges from a drawing of a smiling man doll at one end to a miserable sad man doll in the other. For the arousal dimension, the designs vary from stimulated and alert man doll to a relaxed and calm man doll. The subject should place an “X” on each of the scale drawings or between two subsequent drawings resulting in a scale from 1 to 9. The value “1” represents the lowest score in each dimension, that is, little pleasure/little arousal, whereas the value “9” is the highest score in each dimension, that is, high pleasure/high arousal.

### Memory Tasks

#### Story Recall: Logical Memory

The logical memory test consists of a story used in clinical practice as a tool for verbal memory assessment. The story contains 26 unit-ideas, each one equivalent to an item. The unit-idea may consist of one or more words, e.g., along the story line the name of a person (Ana Soares) is presented and it is one unit-idea. The experimenter reads the story in a loud voice, and the individual is asked to recall immediately after the presentation (immediate recall) and 30 min after the presentation (delayed recall). Subject score consists on the sum of exact recall of all the items, with the maximum of 26 items [WMS-R (52)].

#### Deese–Roediger–McDermott Procedure

This is a recognition test that associates words with neutral and emotional content (7, 53, 54). In this study, were chosen 15 lists (each one contained 15 words) that had the highest rates of false recognition at Stein study. The word lists comprised four positive (music, fruit, sweet, and sleep), four neutral (chair, cold, pen, and high), and seven negative (thief, trash, smoke, needle, grief, pain, and fear) lists.

The presentation order of the words was randomly generated and varied for each subject. The participants were instructed to encode all lists. The words of each list revolve around a theme in which it is strongly associated. These words were termed critical keywords [e.g., *smoke* (critical word), for which associated words that belong to a common theme are cigarette, puff, blaze, billows, pollution, ashes, cigar, chimney, fire, tobacco, stink, pipe, lungs, flames, and stain] that were the related lures.

The recognition task was carried out immediately after presentation of the 15 lists. The recognition task consisted of 90 words, of which 45 of them were targets, 15 related lures, and 30 unrelated lures. The targets are the studied words in the original material taken from positions 1, 8, and 10 of the lists *(hit rates*); the related lures were words not presented in the original material but represent the semantic essence of each of the lists (*false alarm*); and the unrelated lures were words not presented in the original material that have no semantic relationship with them (*response bias* measured by items intrusions) (55). The subjects were asked to circle the words, presented in a sheet of paper that they thought to have seen before. If they circle a *target*, the measure is considered a “*hit rate*” and if they circle a *related lure*, it is considered a “*false alarm*.” All subjects received the same test sheet. The complete lists are shown in Table 1.

**Table 1:** **Within-list words presented in this order**.

DRM task – English equivalents of associated words lists used in this study, originally in Portuguese							
**Negative**							
**Thief**	**Trash**	**Smoke**	**Needle**	**Hurt**	**Pain**	**Fear**	

Robbery	Dirt	Cigarette	Seam	Sadness	Suffering	Dark	
Prison	Recycling	Addiction	Row	Feeling	Bruise	Death	
Assault	Stink	Cancer	Thin	Tear	Loss	Loneliness	
Police	Leftover	Harmful	Needle tip	Rancor	Crying	Anguish	
Jail	Tin	Lung	Pierce	Deception	Nuisance	Panic	
Dishonest	Bag	Drug	Injection	Disillusion	Wound	Scare	
Robber	Pollution	Horrible	Syringe	Frustration	Remedy	Unknown	
Insecurity	Organic	Cigar	Stick	Forgotten	Tooth	Dread	
Money	Dry	Cough	Knitting	Annoying	Head	Violence	
Theft	Waste	Nicotine	Embroider	Bitterness	Missing	Phobia	
Poverty	Problem	Marijuana	Haystack	Mark	Blood	Cry	
Revolver	Rotten	Ash	Machines	Attitude	Accident	Terror	
Escape	Basket	Lighter	Metal	Infidelity	Analgesic	Trauma	
Corruption	Disposable	Swallow	Yarn	Melancholy	Unbearable	Tremor	
Delinquent	Collection	Tobacco	Nailing	Shortage	Desperation	Fear	

**Positive**				**Neutral**			
**Music**	**Fruit**	**Sleep**	**Sweet**	**Chair**	**Cold**	**Pen**	**High**

Sound	Healthy	Dream	Sugar	Sit	Ice	Write	Low
Dance	Apple	Bed	Yummy	Table	Winter	Ink	Building
Disk	Vitamin	Rest	Chocolate	Wood	Coat	Blue	Large
Rhythm	Banana	Wake	Flavor	Object	Snow	Paper	Long
Melody	Strawberry	Nightmare	Honey	Convenience	Blanket	Helpful	Edifice
Singer	Juice	Essential	Ice cream	Room	Sweater	Notebook	Sky
Lyrics	Orange	Accompanied	Delight	Swings	Blouse	Communication	Imposing
Radio	Mature	Lying	Candy	Furniture	Coziness	Ballpoint	Far away
Guitar	Pear	Energy	Snack	Backrest	Temperature	Case	Distant
Instruments	Nutritious	Early	Dainty	Class	Heat	Proof	Stature
Notes	Watermelon	Nap	Diabetes	Upholstered	Chill	Scratch	Difficult
Harmony	Juicy	Hammock	Fat	Support	Rain	Signature	High
Listen	Grape	Silence	Caries	Armchair	Wool	Cap	Size
Voice	Salad	Afternoon	Pie	Bench	Fireplace	Letter	Thin
Electric guitar	Peach	Gape	Taste	Decoration	Soup	Material	Giant

*Words were translated to English; for the words in Portuguese, see Stein et al. (54). The lists were randomly presented, so the order of presentation changed from subject to subject*.

#### DRM–IAPS Task

It was adapted by Pinto et al. (11) and based on the DRM test commonly used in false recognition studies (7, 56). The building set of associated images was extracted from the *International Affective Picture* (IAPS) (9) database, which is a widely used database that contains hundreds of complex, realistic pictures. The IAPS was standardized for the Brazilian population (10). The images contain hundreds of color photographs that can induce many emotional states. The two primary dimensions are affective valence (ranging from pleasant to unpleasant) and arousal (ranging from calm to excited). Of the 20 blocks, 5 are neutral (abstract figures, mushrooms, men, housewares, and clouds), 8 are positive (food, sports, extreme sports, sex, couples, mountain, babies, and family), and 7 are negative (guns, fierce animals, snakes, mutilated bodies, disfigured faces, violence, and car accidents). Each photograph had the level of arousal and valence determined in previous studies (10) with means calculated for each of the 20 blocks.

The recognition task consisted of two stages:

*Study phase (encoding)*: 120 photos were grouped into 20 sets of 6 thematically related pictures. Each set had a main theme (e.g., sex, guns, or abstract figures). The participants were shown pictures ordered by sets photo by photo, for 3 s each with an inter-stimulus interval of 1 s.
*Test phase (recognition)*: within each six-picture set, three pictures were presented again (*targets*), two were new pictures (*related lures*), and one picture was not related to any of the sets from test phase but had the same valence and awareness set level (*unrelated lures*). The recognition task was performed 1 h later, and the participants were shown 120 pictures (60 targets, 40 related lures, and 20 unrelated lures) in a random order. Each picture was presented for 4 s, with a 3-s inter-stimulus interval. The participants were asked to say, for each picture, “Yes” if they thought they had seen it before (in the study phase) or “No.” The picture sets had been standardized for thematic, perceptual, and emotional similarity with a large, representative sample (11).

This method of organizing stimuli into thematically related sets was inspired by previous research with words, which produced robust false recognition effects (23). As with the word lists routinely used in false memory studies (7, 56), the picture sets used here had been standardized for thematic, perceptual, and emotional similarity with a large, representative Brazilian sample (11). In addition, pictures in each set were ranked such that two pictures with a similar score in each set theme were chosen as related lures (to increase the chances of eliciting false recognition responses).

### Memory Data Analysis

This was a randomized, double-blind, and placebo-controlled study. BMI, age, education, IQ, anxiety level, and SAM were analyzed by one-way ANOVA. Memory tasks (DRM and DRM–IAPS) were analyzed with a three-way ANOVA (2 × 2 × 3) with the factors of group (placebo/sulpiride), item (target/lures), and valence (positive/negative/neutral) using Fisher *post hoc* tests. Story recall (WMS-R) was analyzed with a repeated-measures ANOVA. Partial eta-square $\left(\eta_{\text{p}}^{2}=\text{SSeffect}/\text{SSeffect}+\text{SSerror}\right)$ were performed to evaluate the effect size (approximately 0.01 – small, approximately 0.06 – medium, and >0.14 – big effect) (57, 58). Differences were considered significant when *p* < 0.05.

## Results

The participant characteristics were not significantly different between groups (Table 2).

**Table 2:** **Participant demographics**.

Variable	Sulpiride	Placebo	*p*-value
	Mean (SD)	Mean (SD)	
Participant characteristics			
Age (years)	24.58 (4.19)	23.92 (4.70)	0.717
Body mass index (kg/m^2^)	24.56 (1.45)	24.32 (3.57)	0.832
Education (years)	15.92 (1.45)	14.92 (2.78)	0.289
Raven’s matrices (percentile)	88.00 (16.69)	86.08 (15.68)	0.774

*No. of male participants: 12/group. Number is expressed as mean ± SD*.

**p* < 0.05*.

### Mood Effects (STAI)

There was no effect of group on anxiety (STAI-state) before [tpre_(22)_: 0.19; *p* = 0.85] or after the recognition task [tpost_(22)_: −0.49; *p* = 0.62] (Student’s *t*-test). There was no evidence of major differences in anxiety between sulpiride and placebo participants (Table 3).

**Table 3:** **Sulpiride effects on anxiety and episodic memory recall**.

Variable	Sulpiride	Placebo	*p*-value
	Mean (SD)	Mean (SD)	
State-trait anxiety inventory			
STAI_state (pre)	31.83 (4.06)	32.33 (8.04)	0.850
STAI_state (post)	35.08 (4.85)	37.75 (7.87)	0.622
Episodic memory performance			
Story recall (number of unit-idea recalled)			0.07
Immediate	14.33 (3.23)	17.50 (3.55)	
Delayed	13.33 (2.74)	15.25 (4.18)	

*Maximum numbers of items in the story recall = 26; number is expressed as mean ± SD*.

**p* < 0.05*.

### Self-Assessment Manikin

Differences between groups were observed for emotional evaluation of stimuli, specifically the subjective judgment of *pleasure* on the block about *sex* (positive valence) [*F*_(1,22)_ = 6.77; *p* = 0.016] and about *disfigured faces* (negative valence) [*F*_(1,22)_ = 5.25; *p* = 0.032]. The sulpiride group judged the block about *sex* as being more pleasant and the block about *disfigured faces* as more unpleasant than did the placebo group.

Regarding subjective feelings of arousal, the sulpiride group judged the block about mutilated bodies (negative valence) as producing a higher level of arousal than did the placebo group [*F*_(1,22)_ = 5.06; *p* = 0.035].

### Story Recall

The repeated-measures ANOVA revealed no differences between the groups [*F*_(1,22)_ = 3.57; *p* = 0.07], but demonstrated that there were differences in time [*F*_(1,22)_ = 13.64; *p* = 0.001], with no interaction between both factors [*F*_(1,22)_ = 2.01; *p* = 0.16] (Table 3).

### DRM (% Word Recognition)

**Figure 1:**
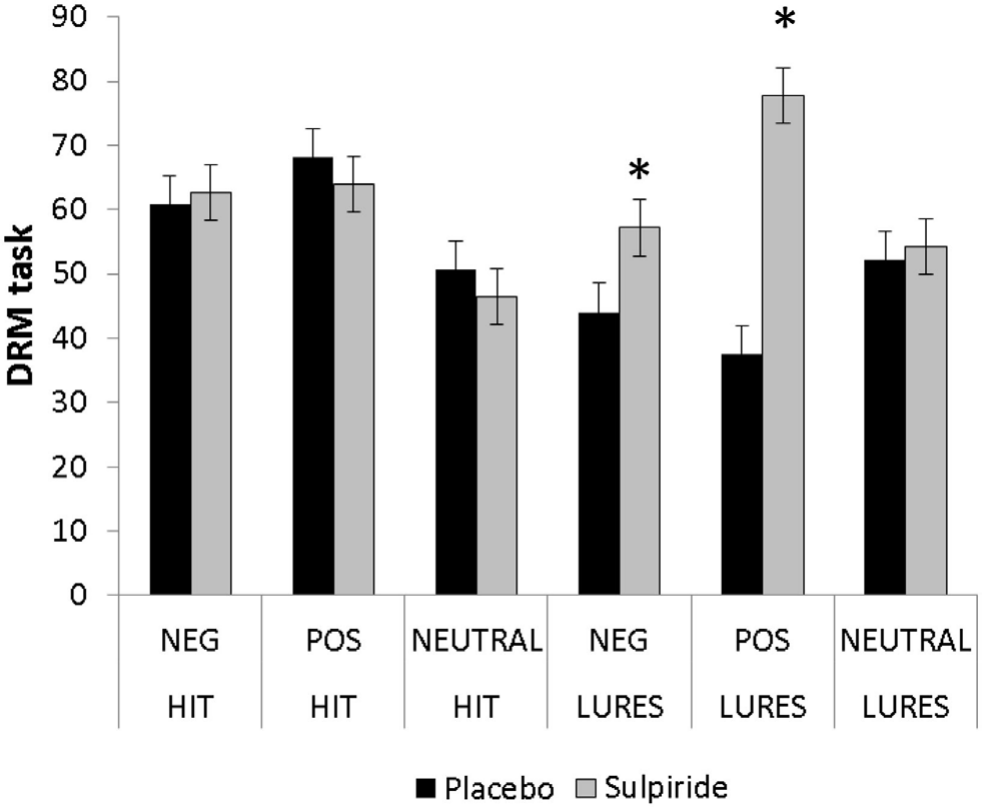
**Percentage of true and false recognition in the DRM paradigm comparing the sulpiride and placebo groups**. Error bars are SEMs; *p < 0.05.

The ANOVA (2 × 2 × 3) design revealed an effect for the three-way interaction [*F*_(2,44)_ = 4.17; *p* = 0.022; $\eta_{\text{p}}^{2}=0.16$] (Figure 1). Figure 1 shows the three-way effect between groups. Strong differences were found for lures on positive lists (*p* = 0.0004). Moreover, higher false alarm rates were found in the sulpiride group than in the placebo group for all valences (Table 4).

**Table 4:** **Percentage of true and false recognition in DRM–IAPS and DRM tasks**.

**Groups**	**Hit rate (%)**			**False alarm rate (%)**		
	
	**DRM paradigm**					
	**Positive lists**	**Neutral lists**	**Negative lists**	**Positive lists**	**Neutral lists**	**Negative lists**
	
Placebo	68.06 (4.79)	50.69 (8.24)	60.71 (5.21)	37.50 (6.53)	52.08 (7.82)	44.05 (4.11)
Sulpiride	63.89 (6.01)	46.53 (6.11)	62.70 (5.37)	77.08 (6.50)*	54.17 (6.02)	57.14 (7.25)*

	**DRM–IAPS paradigm**					
	**Positive pictures**	**Neutral pictures**	**Negative pictures**	**Positive pictures**	**Neutral pictures**	**Negative pictures**
	
Placebo	88.89 (2.68)	84.44 (2.07)	91.67 (2.12)	28.65 (6.08)	45.00 (6.22)	35.12 (5.73)
Sulpiride	89.24 (2.88)	85.00 (2.48)	91.27 (3.09)	43.23 (6.08)^#^	53.33 (5.55)	59.52 (6.72)*

Data are expressed as mean ± SEM; *p < 0.05, ^#^*p* = 0.07. Hit rate: to say “yes” to target; false alarm rate: to say “yes” to related lures.

*DRM: **positive lists**: 12 targets and 4 related lures; **neutral lists**: 12 targets and 4 related lures; and **negative lists**: 21 targets and 7 related lures*.

*DRM–IAPS: total number of blocks: 20 (being 8 positive, 7 negative, and 5 neutral blocks). **Positive pictures**: 24 targets and 16 related lures; **neutral pictures**: 15 targets and 10 related lures; and **negative pictures**: 21 targets and 14 related lures*.

The groups had different false recognition for the unrelated lures on positive lists [*F*_(1,22)_ = 4.76; *p* = 0.04] and negative lists [*F*_(1,22)_ = 9.96; *p* = 0.004]. As expected, no differences between the groups were found on the neutral lists [*F*_(1,22)_ = 2.92; *p* = 0.10] (Table 5).

**Table 5:** **Percentage of false recognition of unrelated lures DRM–IAPS and DRM tasks**.

**Groups**	**False recognition of unrelated lures (%)**					
	**DRM paradigm**			**DRM–IAPS paradigm**		
	**Positive lists**	**Neutral lists**	**Negative lists**	**Positive pictures**	**Neutral pictures**	**Negative pictures**

Placebo	6.67 (2.84)	5.88 (2.29)	17.71 (2.86)	11.46 (3.91)	5.00 (2.61)	7.14 (2.78)
Sulpiride	21.67 (6.26)*	14.22 (4.31)	39.58 (6.31)*	10.42 (2.08)	3.64 (2.34)	8.33 (2.76)

*Number of blocks: **DRM–IAPS** – positive pictures: 8; neutral pictures: 5; and negative pictures: 7. **DRM** – positive lists: 5; neutral lists: 7; and negative lists: 8*.

*Data are expressed as mean ± SEM*.

*p < 0.05.

### DRM–IAPS (% Pictures Recognition)

**Figure 2:**
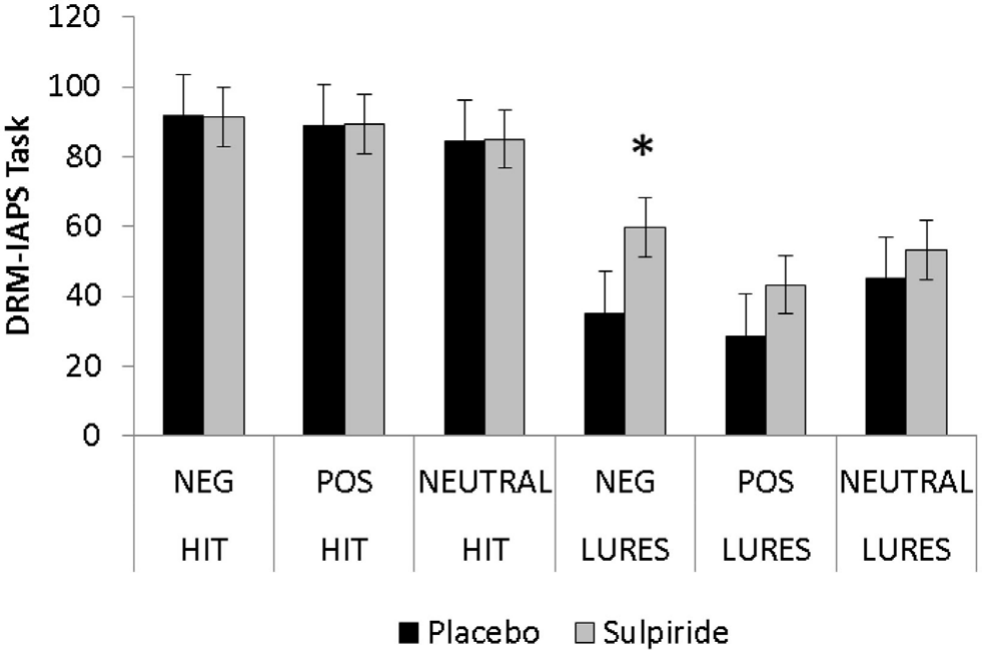
**Percentage of true and false recognition in the DRM–IAPS paradigm comparing the sulpiride and placebo groups**. Error bars are SEMs; *p < 0.05.

The ANOVA (2 × 2 × 3) design revealed an item × valence interaction [*F*_(2,44)_ = 114.47; *p* = 0.001], as well as a three-way interaction (group × item × valence) [*F*_(2,44)_ = 4.95; *p* = 0.01] (Figure 2).

The groups performed similarly on true memories (*p*-values >0.05). Interactions between items and groups were found for false recognition of negative pictures (*p* < 0.05) and approached significance on positive pictures (*p* = 0.07) but not on false recognition of neutral stimuli (*p* = 0.29) (Table 4).

No differences between groups were found on false recognition of unrelated lures on positive pictures [*F*_(1,22)_ = 0.05; *p* = 0.82], negative pictures [*F*_(1,22)_ = 0.09; *p* = 0.76], or neutral pictures [*F*_(1,22)_ = 0.001; *p* = 1.00] (Table 5). In our study, both groups similarly recognized unrelated lures as not seen before in the DRM–IAPS (*p* > 0.05) but not in the DRM task (*p* < 0.05) (Table 5).

## Discussion

The present study showed that dopamine D_2_ receptor blockade by sulpiride, a dopamine D_2_ receptor antagonist, could specifically affect incorrect responses in recognition memory tasks. Sulpiride increased false recognition of related lures (false alarms in both DRM and DRM–IAPS tasks) and also increased false recognition of unrelated lures (intrusion) on DRM task. These increased intrusions were observed just on DRM but not DRM–IAPS, possibly because visual items from DRM–IAPS present more distinctiveness than verbal ones.

Effects on true memory were not observed either on story recall, DRM, or DRM–IAPS tasks. Perhaps, false recognition measures are sensitive to the drugs’ effects than the standard hit rate measures. Ballard et al. (55) demonstrated that amphetamine did not affect emotional and non-emotional true memories but increased false recognition and recall intrusions, an interesting result because amphetamine is a non-selective catecholaminergic agonist. It seems that the mechanisms underlying the false memories are more susceptible to the action of drugs than true memories. On the contrary, Howe et al. (59) proposes that false memories are stronger than true ones; whereas true memories decline over time, false memories actually increase. Howe argued that it may be due to the different ways which they are formed. False memories tend to be *self-generated* as thought or imagination (generated by internal semantic activation). On the contrary, true memories are information that come from outside world and are often *out-generated* (words in a list or images used during the experimental task). Thus, in spite we agree with the above-mentioned authors about the different nature of true and false memories and its vulnerabilities; based on our current findings, we propose that false memories are more susceptible to drug effects than the true ones. The mechanisms underlying such differences, however, still remain to be elucidated.

According to some authors, failures during two processes may contribute to cause the DRM illusion: activation and monitoring processing (8, 56). Activation process failures may be due to increasing interference by overlapping features of memory traces, thus creating confusion between stimuli. Monitoring is described as any memory editing or decision process that helps to determine the origins of the activated information. Failures at monitoring process may also contribute to false recognition, which involves wrong diagnostic decisions to qualify or disqualify information as true (56). Therefore, the activation–monitoring framework (8) predicts that activating similar emotional reactions by related lures or a failure to monitor encoding or retrieval processes determine the propensity to create false memories. It suggests that the increased false recognition observed in the sulpiride group may have been due to the dopamine D_2_ receptors role in activating/monitoring processes. One possibility is that sulpiride produces impairment on monitoring processing leading the subjects to confound studied and non-studied items.

The dopaminergic system is widely recognized as playing an important role in executive function and working memory and therefore in monitoring mechanisms of cognitive and behavioral functions. Dopamine D_1_ receptors activation of prefrontal cortex is necessary for working memory as seen in monkeys (60, 61) and humans (62, 63). However, not only dopamine D_1_ receptors are involved in working memory but also dopamine D_2_ receptors. It has been shown that D_2_ receptor antagonists impair working memory and executive functions (34–36), as well as attention (64–66) in humans.

The prefrontal cortex is the primary mediator of working memory, but the distribution of dopamine D_2_ receptors in this area is limited, but abundant in striatum and hippocampus (67). These authors found that dopamine D_2_ receptors in the hippocampus were associated with frontal lobe functions as executive functions. Therefore, the deficits observed in the present study may have been due to the actions of sulpiride elsewhere in the brain, e.g., hippocampus or striatal dopamine D_2_ receptors. Thus, the hippocampus–prefrontal interaction or fronto–striatal–thalamic circuit may be assumed as the sites of action of dopamine D_2_ receptor modulation of false memories. Further studies are clearly necessary to elucidate these issues.

Moreover, dopamine participates in decision-making processes, particularly response selection in specific circuits in which the dorsal striatum is involved with reward-related actions (45). Failures in this process may have contributed in the generation of false memories.

In the present study, sulpiride did not affect remembered emotional and non-emotional stimuli but increased false recognition only for emotionally charged items (positive and negative) and not neutral ones. This suggests that the dopaminergic system plays a role in emotional aspects of stimuli processing, more specifically through emotional valence modulating trace memory strength through the amygdala. Evidence that dopamine modulates the response of the human amygdala to sensory information was found in an fMRI study of Parkinson’s disease (46). In this neuroimaging study, a robust bilateral amygdala response to fearful stimuli (faces) was observed in the control group, but this response was absent in patients during the hypodopaminergic state. Consonant with this idea, the SAM results of the present study revealed a change in the subjective judgment of the pleasure dimension of blocks (sex/disfigured faces).

The current SAM results also revealed a change in subjective judgment of arousal (mutilated bodies). Arousal has the capacity to modulate memory at many stages of stimuli processing including perception, encoding, and retrieval, playing a critical role in memory by regulating the focus of attention and consolidation (68). Studies with animals have revealed that the amygdala is the key structure for increased memory dependent on the hippocampus during arousal events (69, 70). A recent study suggested a functional role of D_1_ and D_2_ dopamine receptors in the overall potentiation of amygdala response to emotional loaded stimuli in humans (71).

The dopamine potentiates the amygdala response to emotionally loaded stimuli by two ways: (1) increasing the excitatory sensorial input (through dopamine D_2_ receptors stimulation) and at the same time, it (2) attenuates the inhibitory input from the prefrontal cortex through dopamine D_1_ receptor stimulation. In addition, the salient stimulus (emotionally loaded) increases dopamine concentration in basolateral amygdala and thus the local neuronal excitability (through dopamine D_2_ receptors) (47). Thus, based on that, the current results may indicate that sulpiride oral administration increased false recognition by reducing the distinctiveness of emotionally loaded stimuli.

The present study is limited by the small sample size, although efforts were taken to control the similarity of the groups (well-matched according to age, BMI, intelligence, mood baseline, and years of study) at the beginning of trial. Individual variability in response to the drug and the subjective effects cannot be ignored. Another limitation to be considered is that both DRM and DRM–IAPS tasks were applied in laboratory conditions that only partially represent real life, as autobiographical memories are much more complex and include a whole recollection of an event (personal relevance, emotional salience, social context, and perceptual details that differ among people) (56). Nevertheless, DRM illusions are considered indicative of false memory and thus may be representative of autobiographical memory (6, 26, 56, 72–74). Future studies might address the role of dopaminergic neurotransmission in false memory on each single memory stage (encoding, consolidation, or retrieval).

To summarize, we envisage two mechanisms to explain the increased false memories of the sulpiride group, one involving working memory/executive functions through corticostriatal as well as hippocampus–prefrontal D_2_ dopaminergic modulation (67, 75) and the other through D_2_ dopaminergic modulation of the response of the amygdala to emotionally loaded stimuli (43, 47, 71). These possible mechanisms fit quite well the two-process view of the causes of false memories, the activation/monitoring failures (7, 56).

## Author Contributions

All the authors contributed substantially to the planning of theoretical interpretation and writing the manuscript. JG was the psychiatrist responsible for the clinical trial. RG was responsible for performance of the experiment, having applied all the tests to the subjects with collaboration of RR. AS collaborated in planning and statistical analysis. LC and OB helped with interpretation of data and revising the manuscript.

## Conflict of Interest Statement

The authors declare that the research was conducted in the absence of any commercial or financial relationships that could be construed as a potential conflict of interest.
